# The Indole Pulse: A New Perspective on Indole Signalling in *Escherichia coli*

**DOI:** 10.1371/journal.pone.0093168

**Published:** 2014-04-02

**Authors:** Hannah Gaimster, Jehangir Cama, Silvia Hernández-Ainsa, Ulrich F. Keyser, David K. Summers

**Affiliations:** 1 Department of Genetics, University of Cambridge, Cambridge, United Kingdom; 2 Cavendish Laboratory, University of Cambridge, Cambridge, United Kingdom; University of Manchester, United Kingdom

## Abstract

Indole has diverse signalling roles, including modulation of biofilm formation, virulence and stress responses. Changes are induced by indole concentrations of 0.5–1.0 mM, similar to those found in the supernatant of *Escherichia coli* stationary phase culture. Here we describe an alternative mode of indole signalling that promotes the survival of *E. coli* cells during long-term stationary phase. A mutant that has lost the ability to produce indole demonstrates reduced survival under these conditions. Significantly, the addition of 1 mM indole to the culture supernatant is insufficient to restore long-term survival to the mutant. We provide evidence that the pertinent signal in this case is not 1 mM indole in the culture supernatant but a transient pulse of intra-cellular indole at the transition from exponential growth to stationary phase. During this pulse the cell-associated indole reaches a maximum of approximately 60 mM. We argue that this is sufficient to inhibit growth and division by an ionophore-based mechanism and causes the cells to enter stationary phase before resources are exhausted. The unused resources are used to repair and maintain cells during the extended period of starvation.

## Introduction

Indole is a signalling molecule secreted by over 85 species of bacteria including *Escherichia coli* [1]. It is produced by the enzyme tryptophanase (TnaA) that converts tryptophan into indole, pyruvate, and ammonia [2]. *E. coli* makes very little indole during exponential growth. However tryptophanase expression is strongly up-regulated by the stationary phase sigma factor RpoS [3], so indole production rises as cells approach stationary phase [4]. The final concentration of indole in stationary phase culture depends upon the amount of tryptophan in the growth medium. In LB medium free tryptophan can range between 0.5–1 mM, and supernatant concentrations of indole also typically reach 0.5–1 mM [5].

Indole has diverse signalling roles including modulation of biofilm formation, virulence and stress responses [6]–[8]. It can also act as an interkingdom signal and has been shown to affect gene expression in human enterocytes [9] and membrane potential in mitochondria [10]. In each of these cases the system is responding to relatively low (<1 mM), persistent concentrations of indole.

Recent work has revealed a role for indole in plasmid stability. Dimerisation of the multicopy plasmid ColE1 is a well-characterised cause of plasmid instability [11]. The accumulation of plasmid dimers in a cell triggers the synthesis of a 70nt regulatory RNA, Rcd, encoded within the plasmid *cer* site [12]–[13]. Rcd expression leads to inhibition of cell division and it is suggested that the activation of this checkpoint gives sufficient time for the dimers to be converted to monomers by site-specific recombination at the plasmid *cer* site [11].

The target of Rcd is tryptophanase [4] and Rcd binding reduces its K_m_ for tryptophan, stimulating indole production. Since the addition of indole to the culture medium of growing cells was shown reversibly to arrest cell division [10], it was proposed that indole production by Rcd-activated tryptophanase was the mechanism by which the Rcd checkpoint blocks cell division.

Indole has been shown to arrest bacterial growth and cell division by acting as an ionophore and making the cytoplasmic membrane permeable to hydrogen ions [10]. The consequent loss of membrane potential prevents the function of the MinCDE system and localization of the FtsZ ring that is required for cell division. However, while this effect is seen at indole concentrations of 3–5 mM, the maximum concentration of indole in a culture supernatant culture is typically 0.5–1 mM. Unsurprisingly, therefore, it has been unclear whether the effects of 3–5 mM indole upon bacterial cells are biologically relevant.

Here we report a novel mode of indole signalling during *E. coli* stationary phase entry that is important for viability in long-term stationary phase. We show that during the transition from exponential to stationary phase cells transiently experience a very high (>50 mM) concentration of indole and that this pulse is necessary for long-term stationary phase viability. This first example of a “pulse signalling” mechanism expands the repertoire of indole effects on bacteria to include ionophore-mediated effects on cell division and growth. We speculate that it may prove to be of widespread significance.

## Materials and Methods

### Strains and culture conditions

*E. coli* BW25113, W3110, BW25113 Δ*tnaA* (Kanamcyin resistant: Km^R^) and W3110 Δ*tnaA* were obtained from the Keio collection [14]. Cells were cultured routinely in Luria Bertani (LB) medium at 37°C, with shaking at 120 rpm. Overnight cultures were diluted to OD_600_  =  0.05 and allowed to grow for 2 hours before samples were removed for subsequent assays. Where required, indole (dissolved in ethanol) was added; ethanol alone was added to controls where appropriate. To assess colony forming units (CFU), cultures were diluted appropriately and spread onto LA plates. These were incubated at 37°C and colonies counted the following day.

### Mixed culture experiment

Overnight cultures of BW25113 and BW25113 Δ *tnaA* (Km^R^) cells were diluted into fresh LB medium, in triplicate, to an OD _600_ of 0.05 at an initial ratio of 99:1 (BW25113 cells to BW25113 Δ *tnaA).* These cultures were grown at 37°C, with shaking, for 24 hours. At 0, 2, 4, 6, 8, and 24 hours the samples were diluted appropriately and were plated onto both LA and LA + kanamycin plates. This allowed the proportion of Δ *tnaA* cells in the population to be determined.

### Kovacs Assay

Kovacs assay is a commonly-used technique in the field which gives consistent results in different laboratories [4]–[5]. The cell associated indole concentrations presented in this report (to a maximum of 60 mM) are much higher than culture medium concentrations measured previously using the Kovacs assay. However, these are *calculated* cell-associated values. The “raw” assay underpinning the calculation is in the conventional, linear range for the assay.

To assay indole in culture supernatants, a sample (1 ml) from a growing culture was removed, the OD_600_ measured and cells harvested by centrifugation at 11337 x g for 15 seconds (Eppendorf Minispin microfuge). The supernatant was removed and assayed: 300 μl of Kovacs Reagent (10 g of *p*-dimethylamino-benzaldehyde dissolved in a mixture of 50 ml of HCl and 150 ml of amyl alcohol) was added to the supernatant and incubated for 2 minutes. A 50 μl portion was removed and added to 1 ml of HCl-amyl alcohol solution (75 ml of HCl and 225 ml of amyl alcohol). The absorbance at 540 nm was measured (Gene Quant 1300, GE Spectrophotometer). The concentration of indole in the supernatant was calculated using a calibration curve.

To assay indole within the cell pellet, a modified method was used. A sample (1 ml) from a growing culture was removed, the OD_600_ measured and cells harvested by centrifugation at 11337 x g for 15 seconds. The supernatant was discarded and the cell pellet assayed: 300 μl of Kovacs Reagent was added to the cell pellet for 2 minutes. This lysed the cells and allowed the Kovacs reagent to react with indole. This mixture was pipetted into 1 ml LB before a 50 μl portion was removed and added to 1 ml of HCl-amyl alcohol solution. The absorbance at 540 nm was measured (Gene Quant 1300, GE Spectrophotometer). The concentration of indole was calculated using a calibration curve. The samples used to generate the calibration curve contained an appropriate density of indole non-producing cells as well as indole at a known concentration. In addition, the blank for the assay was generated from culture medium containing cells but no added indole. Therefore, any background due to cell debris is taken into account.

Using the concentration of indole obtained from the calibration curve allowed us to determine the number of moles of indole present in the cell pellet *(Ip).* Using the OD_600_ of the culture, we can calculate the total bacterial cell volume *(Vc)* contained in the pellet [15] and hence the apparent cell associated indole concentration *(C_A_)* as shown in equation 1.



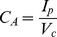
(1)


Calculations of the apparent cell associated indole concentrations based on cell pellet assays may overestimate the indole concentration. This is due to the inclusion (in the *Ip* term) of indole dissolved in the culture supernatant and trapped in the cell pellet. The calculation outlined below allows us to correct for this error.



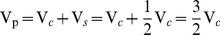
(2)


Equation 2 states that that the volume of the pellet is equal to the sum of the volume of the trapped supernatant and the volume of the cells. Reference 16 states that in a cell pellet, approximately 1/3 of the pellet will correspond to trapped supernatant.



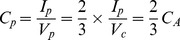
(3)

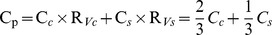
(4)


where C_A_ is the apparent cell associated indole concentration in the pellet, I_p_ is the number of moles of indole in the pellet, V_c_ is the volume of the cells, V_p_ is the volume of the pellet and V_s_ is the volume of the supernatant, C_p_ is the concentration of indole in the pellet, C_c_ is the concentration of indole in the cells and C_s_ is the concentration of indole in the trapped supernatant, R_Vp_ is the volume fraction of the cells in the pellet (R_Vp_ = 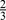) and R_Vs_ is the volume fraction of the supernatant in the pellet (R_Vs_ = 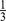), taken from [16].

For example, where C_A_ = 60 mM and C_s_ = 4 mM, so by using Equations 3 and 4 we can estimate C_c_ =  58 mM.

### UV Absorbance Assay of Indole Concentration

Absorbance measurements were carried out using a Varian Cary 300 Bio UV-VIS Spectrophotometer. Unless otherwise stated, chemicals and media were obtained from Sigma Aldrich UK. W3110 *ΔtnaA* cells were cultured overnight at 37°C at 250 rpm in shake flasks containing 30 ml LB medium containing kanamycin (30 μg ml^−1^). Cells were washed by centrifuging the culture for 10 min at approximately 2685 x g (4,000 rpm in a Harrier 18/80 Refrigerated Centrifuge), removing the supernatant and resuspending the pellet in phosphate buffered saline (PBS; prepared by dissolving PBS tablets in an appropriate volume of deionized (Milli-Q) water; this was then autoclaved and sterile filtered (0.22 µm, Millipore) prior to use). After washing the cells were again harvested by centrifugation and re-suspended in the desired amount of PBS. The OD_600_ of the cultures was determined both before washing (in LB) and after washing (in PBS).

One ml aliquots of cells in PBS were transferred to Eppendorf tubes (Axygen, 1.5 ml MaxyClearMicrotubes). Appropriate amounts of 75 mM indole stock solution (in absolute ethanol) were added to obtain the desired supernatant concentrations. A control without indole was also prepared.

After indole addition, the samples were vortexed and the cells were pelleted by centrifugation for 5 minutes at 3500 g (6,000 rpm in a Thermo Scientific Heraeus Fresco 17 Centrifuge). The supernatants (referred to as ‘bacterial supernatants’) were diluted 1∶20 in PBS and their absorbance values measured in the 250–300 nm range. The dilution was required for the absorbance values to lie within the linear range of the spectrophotometer. The indole peak absorbance at 268 nm was determined for all the samples. Background subtraction was applied using the control.

Separately, absorbance curves were measured in the 250–300 nm range for indole solutions (0–2.5 mM) prepared in PBS (after diluting 1∶20 in PBS similar to the steps described above). The indole absorbance peak at 268 nm was measured, background subtraction was performed using the ‘no-indole’ sample and the peak absorbance values were used to plot a calibration curve of Absorbance vs External Indole Concentration.

Using the above calibration curve and the absorbance value measured from the bacterial supernatant, the indole concentration of the bacterial supernatant was determined. Subtracting this from the known external concentration allows us to determine the number of moles of indole present in the bacterial pellet. Using the OD_600_ obtained (in PBS), we can calculate the total bacterial cell volume contained in the pellet [15] and hence the apparent cell associated indole concentration.

### Determination of the *E. coli* lipids-buffer partition coefficient of indole

*E.coli* total lipid extract (Avanti Polar Lipids) dissolved in chloroform was added to a 5 ml vial. The organic solvent was removed by high-vacuum drying for 3–4 hours to give a 5 mg lipid film. Solution A (5 mM indole, Sigma Aldrich, in 15 mM phosphate buffer (PB; pH = 7)) was prepared by adding the appropriate volume of a stock solution of indole in ethanol to the 15 mM PB buffer. The lipid film was dispersed by adding 3.5 ml of solution A and sonicating using an ultrasonic bath (Grant, MXB6, 160W) for around 2 hours at 25°C. In order to separate the lipid and aqueous phases, samples were centrifuged with a Beckman Coulter-Optima max XP Ultracentrifuge at 50,000 x g for 45 min at 25°C. After ultracentrifugation, lipids appeared as a pellet at the bottom of the centrifugation tubes. The aqueous phase was taken carefully with a Pasteur pipette and kept in an Eppendorf tube for the spectrophotometric measurements.

The *E. coli* lipids-buffer partition coefficient of indole was determined using a Varian Cary 300 Bio UV-VIS Spectrophotometer. A calibration curve of absorbance *vs* indole concentration at 268 nm was initially obtained by preparing indole solutions in the 15 mM PB buffer in a range of concentrations of 0–0.2 mM (linear range). The indole concentration of the aqueous phase was determined by measuring the absorption at 268 nm, normalising the values with respect to the ‘no-indole’ and using the calibration curve to estimate the concentration. No-indole samples containing the same quantity of lipids (5 mg) dispersed in PB buffer but lacking indole, were submitted to the same ultracentrifugation and separation process. The concentration of indole in the lipid phase was calculated by subtracting the amount of indole in the aqueous phase from the total amount present in the initial solution A.

The partition coefficient (P) was then calculated using equation [5] [17] where C_i_ is the concentration of indole in solution A, C_w_ is the concentration of indole in the aqueous phase, w_w_ is the weight of the aqueous phase (3500 mg) and w_l_ the weight of the lipid phase (5 mg) in the samples.



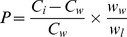
(5)


### Estimation of the volume of the lipid membrane in *E. coli*

The volume of the lipid membrane in *E.coli* (0.04 μm^3^) has been determined using equation [6] and applying simple geometrical calculations. In these calculations, *E. coli* is assumed to have a cylindrical shape. *l* is the length of *E.coli* (1.6 μm) [15], *r* is the estimated radius of the cylinder (0.55 μm) and *d* is the thickness of the lipid bilayer (around 5nm) [18].



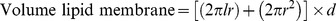
(6)


The volume of the entire *E. coli* is 1.5 μm^3^, so the lipid membrane represents 1/40^th^ of the total cell volume.

### Estimation of the molecular ratio indole:lipid and the weight percentage indole:lipid

The estimation of the molecular ratio indole:lipid (0.4±0.1) has been made using Equation [8] (simplified from Equation [7]) where *d* is the density of the lipids (1000 g L^−1^), V_lipid_ is the volume of the lipid membrane in a single *E. coli* (0.04 μm^3^), M_lipid_ is the molecular weight of *E. coli* total extract lipids (811.5 g mol^−1^), N_A_ is the Avogadro constant (6.023×10^23^), C_indole_ is the concentration of indole added to the supernatant (0.005 mol L^−1^) and P is the *E. coli* lipids-buffer partition coefficient (92.9±24.5).



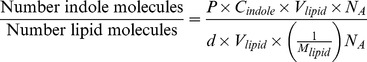
(7)




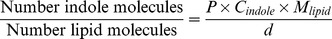
(8)


The percentage in weight indole:lipid has been calculated multiplying Equation [4] by the ratio of molecular weights of indole:lipid, namely (117.2:811.5).

## Results

### Indole production increases rapidly during entry into stationary phase

**Figure 1:**
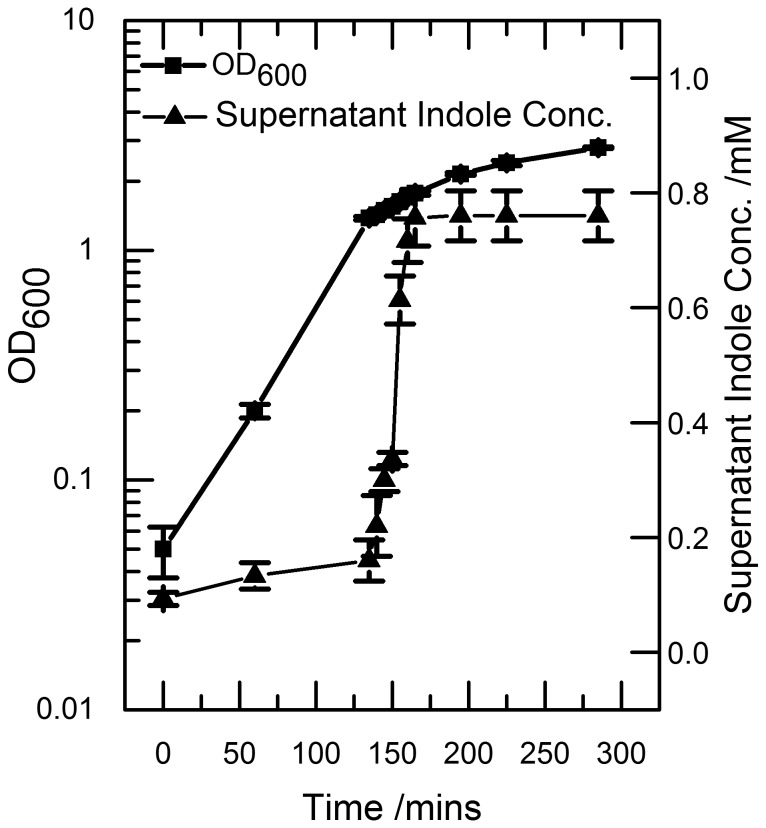
Indole is produced rapidly over a 30 minute period during the onset to stationary phase. A culture of growing BW25113 cells was sampled regularly. The OD_600_ was measured and the samples were centrifuged to remove cells, and the supernatant was assayed for indole using Kovacs assay. Data shown are the mean values ± standard deviation for three independent repeats.

The concentration of indole in the supernatant of an L-broth culture of *E. coli* BW25113 was measured using the Kovacs assay (Fig. 1). Consistent with reports elsewhere [5], the supernatant concentration reached a maximum of 0.7–0.8 mM in stationary phase. The concentration was low in late exponential phase but rose rapidly when the culture reached an optical density (OD _600_) of 1.0–1.5. This corresponded to the period of transition between exponential growth and stationary phase.

The rapid accumulation of indole in the supernatant (5-fold increase over 30 min) reflected an increased production rate *per* cell rather than simply an increase in cell numbers, since the OD_600_ of the culture increased only 1.4-fold during this period.

### Indole production is required for long-term stationary phase viability

**Figure 2:**
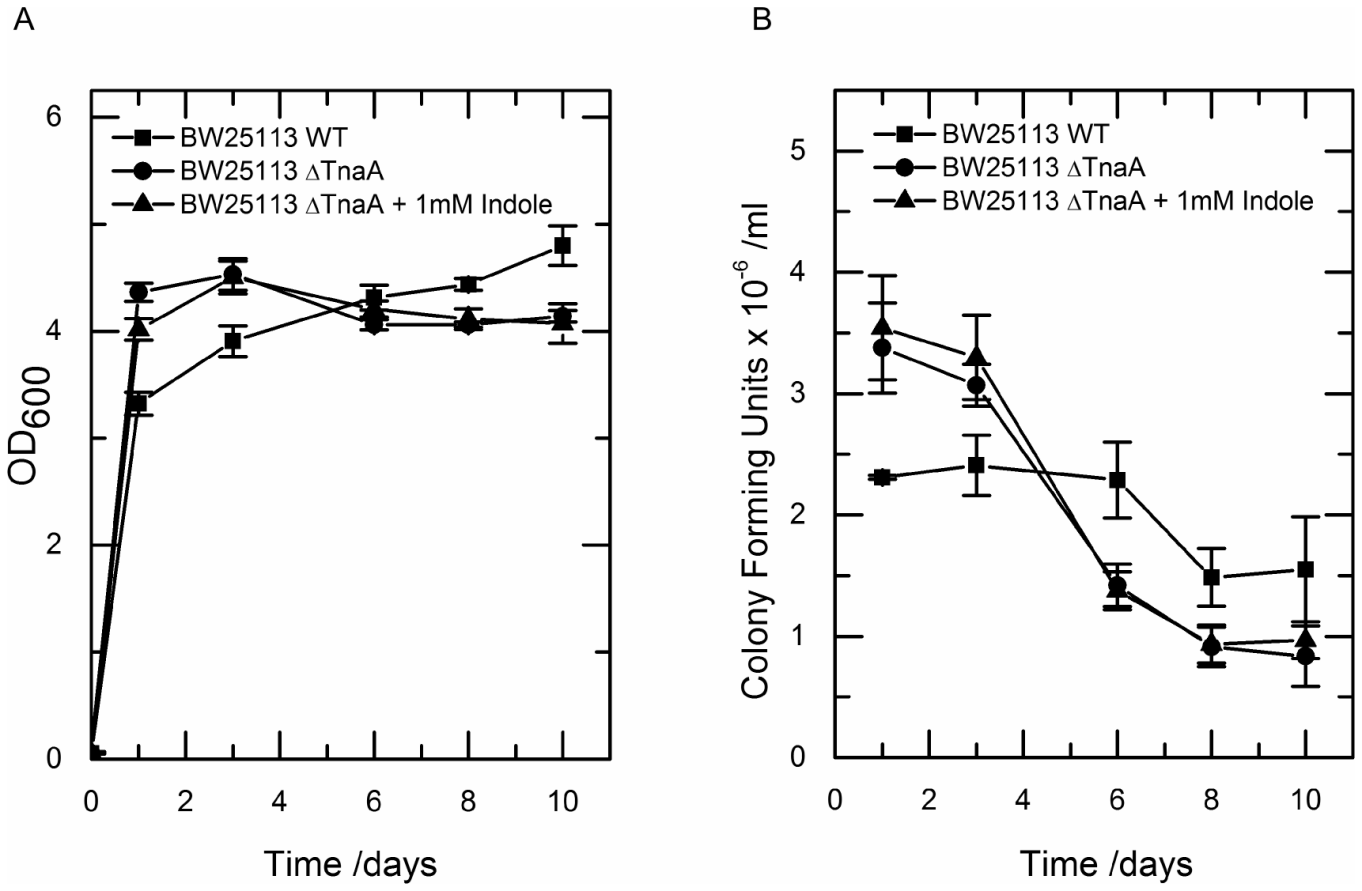
Non indole producing mutants initially grow to a higher density and are more viable than indole producing counterparts, but are significantly less viable in the long term. The density (OD_600_: A) and viability (CFU ×10 ^−6^/ ml :B) of wild-type, mutant and mutant with 1 mM indole added were assessed over 10 days. Data shown are the mean values ± standard deviation for three independent repeats.

In order to assess the functional significance of indole production during the onset of stationary phase, we compared the growth and viability of BW25113 and BW25113 Δ*tnaA* (a tryptophanase knock-out that produces no indole) over 10 days at 37°C. For the first three days the density of BW25113 cultures was significantly lower than BW25113 Δ*tnaA*, implying that indole production inhibits growth during stationary phase entry. However over the subsequent 7 days the OD_600_ of the mutant culture declined, while the density of the wild-type continued slowly to increase (Fig. 2A). Consequently, after 10 days in stationary phase, it was the wild-type culture that had the higher optical density.

Optical density measurements provide an estimate of the number of cells in a culture but not whether these cells are alive or dead. To compare the viabilities of cells in TnaA^+^ and TnaA^−^ cultures, samples were diluted and spread on L-agar plates to estimate viable counts (Fig. 2B). We observed that over 9 days in stationary phase the colony forming units (CFU) in the mutant culture decreased by approximately 75%, compared to the wild-type culture where CFU decreased by less than 30%. One possible interpretation of these data is that indole production causes growth of the wild-type culture to slow before resources are exhausted and the availability of unused resource assists long-term survival in stationary phase.

In an attempt to restore the stationary phase viability of the Δ*tnaA* mutant strain, indole (1 mM) was added to the culture medium when the OD_600_ reached 1.5. This mimicked the rapid accumulation of indole in the culture supernatant of wild-type cells at this point. However, the addition of 1 mM indole had no effect on either the culture density or the long-term viability of the mutant (Fig. 2).

The failure of 1 mM indole to restore the long-term viability of the Δ*tnaA* mutant was initially surprising. However, indole production by wild-type cells during stationary phase entry appears to slow their growth (Fig. 2A), and indole concentrations of below 2–3 mM are known to have little or no effect on growth [4]. A significantly higher (4–5 mM) indole concentration is required to inhibit growth or cell division. Because indole production during stationary phase entry is very rapid, it is possible that a higher concentration of indole exists inside the cells during the production period. If a sufficiently high concentration were reached, this might explain the slowing of growth observed for the wild-type strain.

To test the proposal that it is the internal rather than the external concentration of indole which determines the kinetics of entry into stationary phase, a mixed culture experiment was performed. The culture contained both wild-type and Δ*tnaA* mutant cells, at an initial ratio 99:1. This allowed us to observe the behaviour of Δ*tnaA* cells in the presence of levels of indole that occur in the wild-type culture supernatant. There is a clear prediction that if the kinetics of stationary phase entry are affected by the external level of indole, then all the cells in the mixed culture should enter stationary phase at the same time and the proportion of the Δ*tnaA* mutant cells should remain constant. However if an elevated internal level of indole in the wild-type producer cells regulates stationary phase entry then the wild-type cells will enter stationary phase earlier and the Δ*tnaA* mutant cells will increase their proportion in the population.

Samples were taken from the mixed culture at regular intervals and the proportion of mutant cells was measured. The proportion increased from 0.7±0.4% when the cultures were set up, to 4.4±0.2% at 24 hours. This corresponds to a 6-fold increase in the proportion of mutant cells in 24 hours. Furthermore, the greatest increase in the proportion of mutant cells (a 4-fold increase) occurred between 6 and 8 hours and was therefore after indole was produced by the wild-type cells. This result suggests that resources unused by wild-type cells remained in the supernatant where they were available for use by the mutants.

### Cell associated indole increases rapidly before stationary phase

**Figure 3:**
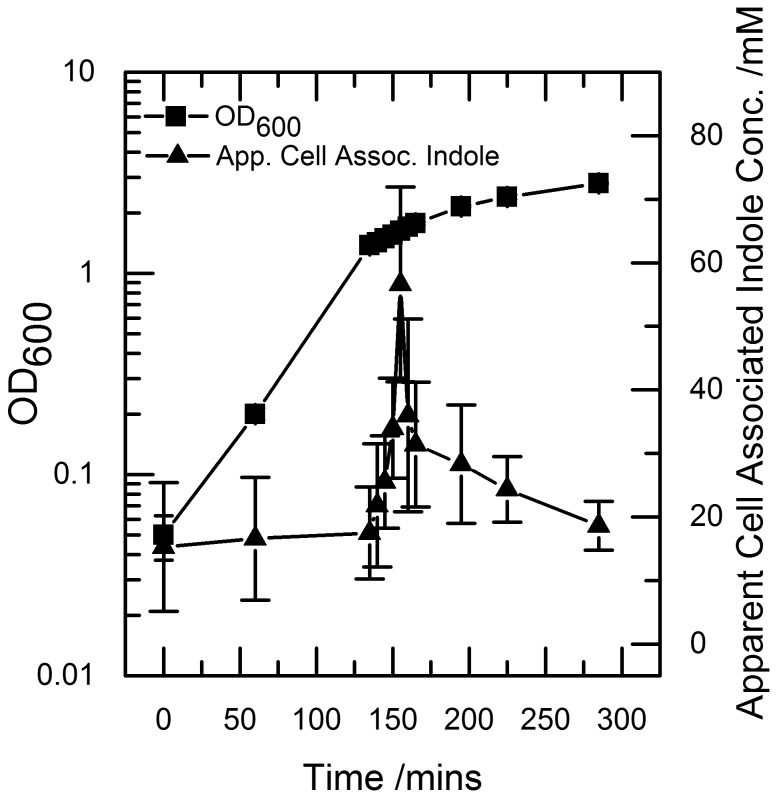
Apparent cell associated indole accumulates rapidly at the onset of stationary phase. A culture of growing BW25113 cells was sampled regularly The OD_600_ was measured and the samples were centrifuged to pellet cells. The resultant cell pellet was assayed for indole using Kovacs assay. Data shown are the mean values ± standard deviation for three independent repeats.

To test the possibility that a high intracellular indole concentration exists during stationary phase entry, we used the Kovacs assay to measure apparent cell-associated indole. Culture samples were centrifuged, the supernatant removed and the cell pellet lysed by the addition of Kovacs reagent. The concentration of indole in the lysate was measured by the Kovacs assay and was converted to the apparent cell-associated concentration using an estimate of *E. coli* cell volume [15]. We use the term ‘apparent’ to acknowledge that the concentration has not, at this stage, been corrected for any contribution from indole in the culture supernatant that is trapped in the cell pellet. The result showed that apparent cell-associated indole increased sharply but transiently during stationary phase entry, reaching a maximum of 60 mM (Fig. 3). Also significant was the observation that the apparent cell-associated indole concentration never dropped below 15 mM and was always substantially higher than the supernatant concentration (Fig. 1).

**Figure 4:**
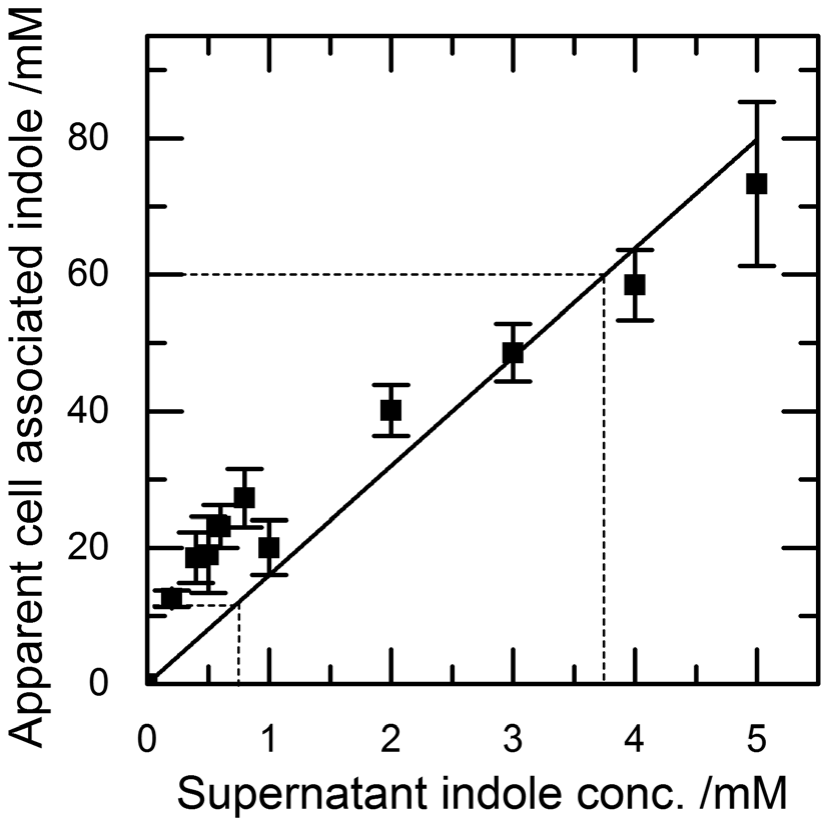
The relationship between apparent cell associated concentrations of indole and supernatant concentrations of indole. Known concentrations of indole were added to the supernatant of stationary phase BW25113 Δ *tnaA* cells, the mixture was vortexed and centrifuged, and the resultant pellet assayed for indole using Kovacs assay. Dotted lines indicates the apparent cell associated indole at the peak of the pulse (60 mM) and the corresponding supernatant concentration and indicates the apparent cell associated indole when cells are in stationary phase and the corresponding supernatant concentration (0.75 mM). Data shown are the mean values ± standard deviation for at least three independent repeats.

The effects of defined concentrations of indole added to the culture medium on the growth and division of *E. coli* are well established but this is not true for cell-associated indole. To help interpret the data of Fig. 3, known concentrations of indole (0–5 mM) were added to stationary phase cultures of a Δ*tnaA* mutant strain. Cells were harvested and the pellet assayed for apparent cell-associated indole (Fig. 4). A supernatant concentration of 0.75 mM (typical of stationary phase cultures of wild-type cells) gave an apparent cell-associated concentration of approx. 20 mM, while an apparent cell associated concentration of 60 mM (the maximum detected during stationary phase entry) resulted from a supernatant concentration of approx. 4 mM.

The apparent cell associated indole concentration can be corrected for the contribution of indole from the culture supernatant. It has previously been estimated that a cell pellet is likely to contain approximately 30% culture supernatant in addition to the cells [16]. We have described in the material and methods a calculation to compensate for the contribution of the extracellular indole in the pellet. In Fig. 4, we see that 4 mM indole in the culture medium gave an apparent cell associated indole of 60 mM. Applying the correction, the actual cell associated indole concentration is shown to be 58 mM rather than 60 mM. Thus, the contribution of indole from trapped supernatant to our apparent cell associated indole concentrations is negligible and well within the experimental errors of our assay.

To confirm that the high concentration of cell-associated indole was not an artefact of the Kovacs assay, the relationship between cell-associated and supernatant indole was investigated by an independent technique. Indole non-producing (TnaA^−^) cells were suspended in medium containing a known concentration of indole and then harvested by centrifugation. The amount of indole removed from the medium by the cells was estimated by using an UV absorbance assay to measure the indole concentration in the medium before and after harvesting (see Materials and Methods for details). When the indole concentration in the medium was 1 mM, a cell-associated concentration of 7±3 mM was calculated (8 repeats). This compares with a value of 17±3 mM when cell-associated indole was measured using the Kovacs assay (Fig. 4). The difference between the two values may be a systematic error due to procedural differences between the assays, however they are still within an order of magnitude of each other and they help confirm that the high cell associated values are not simply an artefact of the Kovacs assay.

### Indole distribution within cells

Apparent cell-associated indole is an average of the indole concentrations in the cytoplasm and in the lipid membranes. To determine the relative indole affinity of these two cell components we measured the partition coefficient (P) of indole between water and *E. coli* total lipid. We obtained a value of log (P)  =  1.95±0.12 (n = 9), which is similar to the calculated value of 2.17 for octanol-water partitioning reported in [19]. Thus indole has an approx. 90-fold higher affinity for lipid than water, due to the hydrophobicity of the aromatic ring of the molecule.

## Discussion

The effects of low (0.5 –1.0 mM) concentrations of indole on bacterial physiology are well established [1]. In addition, recent studies have shown that at higher (4–5 mM) concentrations, indole can regulate bacterial growth and division through its action as a proton ionophore [10]. However, the biological relevance of these effects has been open to question since concentrations above 1 mM are not seen in culture supernatants. In this report we describe a mode of indole signalling that is independent of the supernatant concentration, but is driven by a transient, high concentration of indole inside the cell.

A culture of wild-type *E. coli* makes the transition from exponential to stationary phase earlier than a culture of an indole non-producing mutant. The wild-type also displays higher viability in long-term stationary phase, and a likely explanation is that resources unused for growth remain available to the wild-type for repair and maintenance during the period of starvation as suggested by the mixed culture result. We were unable to restore wild-type behaviour to the mutant by supplementing the culture medium with 1 mM indole. This is perhaps unsurprising when one remembers that 1 mM indole has no detectable effect upon either growth or division of *E. coli* [4]. The indole supplementation experiment was designed so that indole was added to the culture at the same time, and with the same kinetics, as it appears in wild-type culture (Fig. 1). The only difference between the indole-supplemented mutant cells and wild-type cells was that the wild-type cells experienced a transient cell-associated indole concentration of 60 mM (Fig. 3). We therefore conclude that the subsequent difference in growth and viability of the two cultures was due to the presence or absence of this pulse of cell-associated indole. This distinction between the effects of external and cell-associated indole is further demonstrated in the mixed culture experiment. Here, mutant cells that did not experience the indole pulse entered stationary phase later than co-cultured wild-type cells.

The 6-fold increase in indole non-producing cells in the mixed culture experiment also suggests that resources unused by wild-type cells remain in the culture medium and are available for use by other bacteria. This seems a little surprising since the reserve is then available for use by “cheats” that continue to grow until the resource is exhausted. If this is true, we would predict that during long-term stationary phase the mutant cells would maintain their elevated proportion in the mixed culture but that both mutant and wild-type cells would die at the higher rate exhibited by mutant cells in pure culture.

The pulse is well defined and lasts approx. 20 min (Fig. 3). We suggest that this corresponds to a period when indole is being produced faster than it can escape from the cell by diffusion through the membrane. The start of the pulse can then be explained by the RpoS-induced up-regulation of tryptophanase expression as stationary phase approaches [3]. The end of the pulse is more difficult to explain since tryptophanase is presumably still present. However the total amount of indole produced by a culture is limited by the amount of free tryptophan in the growth medium [5], so the end of the pulse probably reflects the time when all available tryptophan has been converted to indole. It is important to note that the pulse duration of 20 min is an average of all cells in the culture. If there is significant population heterogeneity in the time of pulsing, the pulse duration in individual cells may be shorter and the maximum concentration higher than the values we present here.

Interpretation of our data is complicated by the fact that *E. coli* cells, by virtue of their lipid membranes (and possibly other cell components), have a greater affinity for indole than their aqueous surroundings. This is why cell-associated concentrations are always higher than supernatant concentrations, not just during the indole pulse. Since in previous reports the effects of indole on the growth and division of *E. coli* have been determined experimentally by the addition of indole to the growth medium (4, 20), it is helpful to convert apparent cell-associated indole to “supernatant-equivalent concentrations” using the data of Fig. 4. Thus when the apparent cell-associated concentration is 20 mM in late stationary phase, the supernatant-equivalent concentration is approx. 0.75 mM and this is insufficient to have any effect on growth. However, when the apparent cell-associated concentration is 60 mM at the peak of the pulse, the supernatant-equivalent concentration is approximately 4 mM (Fig. 4) and high enough to cause significant inhibition of growth [4]. Thus our data suggest that the magnitude of the indole pulse during stationary phase entry is sufficient to regulate growth and division by the ionophore mechanism described by Chimerel *et al.* [10]. It has previously been reported that at the onset to stationary phase, *E. coli* cells become smaller [21] due to continued elongation and division. Cell size depends on the relative contributions of cell growth and cell division. Indole has been shown to affect both these processes and if growth is inhibited earlier, or more strongly, than cell division by the indole pulse then smaller cells would result.

It is clear from our data that the indole pulse is a non-equilibrium phenomenon. At the peak of the pulse, the concentration of apparent cell-associated indole (60 mM; Fig. 3) is 150-fold higher than the supernatant concentration (0.4 mM; Fig. 1). As the system returns towards equilibrium in stationary phase, the apparent cell-associated concentration (20 mM) exceeds the supernatant concentration (0.75 mM) by less than 30-fold.

The indole:water partition coefficient (log (P)  =  1.95) indicates that the equilibrium concentration of indole in the membrane itself (as opposed to the total cell-associated concentration) will also be substantially higher than the concentration in the surrounding medium. If the lipid membrane accounts for 1/40^th^ of the total cell volume, there will be approximately twice as much indole in the membranes as in the cytoplasm. We estimate the molar indole:lipid ratio as 0.4 (±0.1):1 when 5 mM indole is added to the growth medium (see Materials and Methods for details). However, the molecular weight of *E. coli* lipids is considerably greater than the molecular weight of indole, so it is useful also to express the ratio of indole:lipid as 0.05:1 by mass. Either way, we envisage that the remarkably high concentration of indole in the lipid membrane during the pulse may have a significant direct effect on the properties of membrane proteins, in addition to its effect on ionic permeability.

Does the affinity of *E. coli* lipids for indole explain completely the high cell-associated indole concentrations that we measured under equilibrium conditions (Fig. 4)? To answer this we need to assume that all cell components, other than lipid, have the same affinity for indole as water. We can then calculate the expected cell-associated concentration for any given external indole concentration, based on the lipid:water partition coefficient and the proportion of a cell that is lipid. This calculation predicts that the cell-associated concentration should be approximately 3 times higher than the concentration in the surrounding medium. This is significantly different from the experimental results shown in Fig. 4, where the cell-associated concentration is approximately 15 times higher than the concentration in the growth medium. This seems to imply that some additional component in the cell may have a high affinity for indole. Alternatively, it is possible that the partition coefficient measured *in vitro* may not entirely reflect the situation *in vivo* where the lipid composition may vary and the lipid solubility of indole may be influenced by the membrane charge.
